# A Multisite Study on Knowledge, Perceived Motivators, and Perceived Inhibitors to Precepting Nursing Students within the Clinical Environment in Ghana

**DOI:** 10.1155/2021/6686898

**Published:** 2021-01-18

**Authors:** Nancy Innocentia Ebu Enyan, Sarah Ama Amoo, Christian Makafui Boso, Patience Fakornam Doe, Dianne Slager

**Affiliations:** ^1^University of Cape Coast, School of Nursing and Midwifery, Department of Adult Health, Cape Coast, Ghana; ^2^Cape Coast Teaching Hospital, Cape Coast, Ghana; ^3^Kirkhoff College of Nursing, Grand Valley State University, Grand Rapids, MI, USA

## Abstract

**Background:**

Preceptorship constitutes an important component of the educational process of training nursing students. The purpose of this study was to assess the knowledge, perceived motivators, and perceived inhibitors to precepting nursing students at the clinical placement sites in the Cape Coast Metropolis of the Central Region of Ghana.

**Methods:**

A descriptive cross-sectional study was conducted among 442 nurses and midwives aged 27–56 years with at least three years of work experience. Data were collected with a questionnaire and analyzed using frequency counts, percentages, exploratory factor analysis, and point biserial correlation.

**Results:**

The results indicate that the participants had a high knowledge of preceptorship of up to 91.2% (*n* = 404). A significant proportion of up to 88.2% (*n* = 390) had an intention to precept nursing students in the near future. The three important perceived motivational factors to precepting nursing students were the learning and professional needs of students, helping students to develop skills, and experience and formal recognition of the role of preceptorship. The main perceived inhibitors to engage in a preceptorship role were lack of preparation for the role, lack of support from faculty and nurse managers, and additional work burden. The results further indicate a significant strong positive correlation between experience and professional recognition of preceptorship and the intention to precept nursing students in the near future (*r* = 0.99, *p*=0.037).

**Conclusions:**

The nurses and midwives who participated in the study are knowledgeable about preceptorship and have the intention to precept nursing students. Having enough experience on the job and being formally recognized as a preceptor may motivate these professionals to precept nursing students. However, there are critical perceived barriers that need to be addressed, to enable more nurses and midwives with the desire to precept students to engage in the preceptorship role.

## 1. Background

Preceptorship has been traditionally perceived to be a relationship in which a senior colleague who has a supervisory role grooms a novice colleague to achieve the needed competencies [1]. This process allows the preceptee to seek support and guidance on specific areas of weakness from the preceptor. Previous studies demonstrate precepting to be highly beneficial to nursing students [2, 3]. It is known that preceptorship challenges preceptors to develop skills in their areas of expertise which also facilitates the acquisition of leadership skills and roles [1]. Previous studies have affirmed that precepting involves skills in mentoring, which encompasses formal and informal counseling, guiding, supervising, networking, teaching, advocating, coaching, supporting, sharing, and role modeling [4]. The preceptor is viewed to be skillful and offers training and guidance to newer colleagues who may be less knowledgeable. The process requires a long-term relationship between the preceptor and the preceptee who is often a novice student with much expectations and uncertainties about the nursing profession [1].

The Nursing and Midwifery Council (NMC) in the United Kingdom defined a mentor/preceptor as “a nurse, midwife or specialist community public health nurse who facilitates learning and supervises and assesses students in a practice setting” [5]. Although this definition is from a western country, it fits the description of a preceptor within the Ghanaian context. A representative from the Nursing and Midwifery Council of Ghana (N&MCG) in an earlier study explained that the preceptor's role is expected to focus on collaboration between clinical agencies and training institutions in clinical teaching, organization of clinical conferences, and provision of feedback to students, although there was uncertainty about the extent of implementation [6]. Consequently, preceptors have a responsibility to offer guidance and support to nursing students in the practice setting by creating an environment that enables students to make sense of their experience by applying theory to practice; providing constructive feedback; and facilitating and enhancing reflection on experiences, performance, and practice [7].

Preceptorship in nursing could be described within the framework of Patricia Benner's novice to expert learning theory whereby individuals commence as novices with limited experience but with the needed support, progress to the stage of an advanced beginner. When individuals gain mastery of the expected skills, they become competent and gradually become proficient in recognizing what is important and establishing priorities. Later, they could become experts when they are highly skilled [8]. With this process, nursing students are viewed as novices needing support from faculty and preceptors to achieve competence. Later with experience, they develop more skills in clinical reasoning and judgement to gain mastery and be more proficient and become future experts. In addition, this study was conceptualized within Albert Bandura's Social Learning Theory. The constructs of this study is explained within the intrinsic reinforcement and cognition aspects of the theory [9]. Intrinsic reinforcement considers factors such as motivation and satisfaction. It also emphasizes on cognition and internal thought process, making it suitable for explaining the knowledge, perceived motivators, perceived barriers, and support preceptors required for performing the preceptor role. Consequently, for the novice to attain the desired expertise, preceptors should be knowledgeable, motivated, and supported to function in the preceptor role. It is important that systems are put in place to remove the numerous challenges preceptors might encounter in the performance of the role.

The period of transitioning from a nursing student to an autonomous registered nurse has been described as a stressful time [10]. It is a period where the new nurse needs to be supported by a highly skilled preceptor to enhance critical thinking, refine skills, and develop confidence and autonomy [11, 12]. Preceptors play vital roles in nursing education by helping shape the skills of students and also socialize them into professional nursing roles, thereby facilitating their transition from novice to experts [8]. They also assist the student to link theory to practice. Preceptors serve as a teacher, mentor, leader, and evaluator, as they assist students to integrate into the new work environment. By doing so, students are motivated to stay in the profession, and this increases retention of nurses [3]. The importance of the role of the preceptor cannot be over emphasized.

In Ghana, most nursing schools seem to be operating the preceptorship model in the training of nurses, but this has not been well implemented [13]. In the clinical setting, there is some form of student-preceptor interaction albeit very minimal, and this is mainly undertaken by nurses and midwives who are willing or personally motivated to help students learn. Although the N&MCG expects every professional nurse and midwife to be involved in precepting nursing students, some nurses and midwives decline to be involved in how students learn in the clinical setting. Moreover, in situations where preceptors are available, they are often few and may be overwhelmed and overburdened with the increasing student numbers as well as the competing demands of their daily routines at the workplace. This leads to situations where students either have minimal contact with preceptors during periods of placement or contact with these preceptors may be nonexistent thereby affecting their professional development.

Empirical studies have reported high knowledge of nurses and midwives on preceptorship in developed settings [14, 15]. However, an Ethiopian study found that few nurse educators were knowledgeable about preceptorship [15] although they had good attitudes towards it. Regarding nurses and midwives' motivation for precepting students, willingness to share knowledge, being internally motivated, professional experience [16], and “giving back to the profession” have been cited in the literature [17]. Other important intrinsic motivators include supporting students' learning and professional development [17]. Despite the desire to precept students, some factors including reduced productivity [18] and lack of skills [19] could hinder the process. This study, therefore, sought to assess the knowledge, perceived motivators, and perceived inhibitors to precepting nursing students at the clinical placement sites in the Cape Coast Metropolis of the Central Region of Ghana. Specifically, the study was guided by the following research questions: (1) What is the level of knowledge of nurses and midwives on preceptorship? (2) What are the perceived motivators to precepting nursing students? (3) What are the perceived inhibitors to precepting nursing students? and (4) What support do preceptors need to successfully perform their roles?

## 2. Methods

### 2.1. Study Design and Setting

A descriptive, cross-sectional survey was conducted among 442 nurses and midwives aged 27–56 years working in the Cape Coast Metropolis in the Central Region of Ghana. The Central Region is known as the citadel of education in Ghana. Cape Coast is the capital town of the region, with a host of educational institutions. In the area of nursing and midwifery, three public training institutions run nursing programmes-the School of Nursing and Midwifery of the University of Cape Coast, Cape Coast Nursing and Midwifery Training College, and Ankaful Psychiatric Nursing Training College. Students from these institutions gain clinical learning experience from nurses and midwives working in health facilities within the Cape Coast Metropolis.

### 2.2. Population

The population comprised professional nurses and midwives working in all clinical placement sites in the Cape Coast Metropolis of Ghana. The rationale for including nurses and midwives is that both professionals' precept nursing students in the practice settings. Likewise, in Ghana, some registered nurses have also studied midwifery to be registered midwives. This category of nurses and midwives has dual professional backgrounds. Therefore, this study included both nurses and midwives without clearly delineating the two professions. The population size was estimated to be 1,241 nurses and midwives. Specifically, 806 from the Cape Coast Teaching Hospital [20], 210 from the Ankaful Psychiatric Hospital [21], 79 from the University of Cape Coast (UCC) Hospital [22], 91 from the Metropolitan Hospital, and 55 from the Ewim Polyclinic [23]. Nurses and midwives with at least three years of work experience and working in any of the clinical placement sites were included in the study. These nurses and midwives were assumed to have sufficient knowledge and clinical competence to engage in clinical teaching. Nonetheless, nurses and midwives with less than three years of work experience and those pursuing their national service were excluded because they may not have the required competencies and knowledge to be involved in clinical teaching or precepting students. Again, those on any form of leave did not participate in the study. It is worth mentioning that the study participants had different levels of education and experience, but they were all included in the study because we were also interested in their intention to precept nursing students as well as the perceived motivators and perceived barriers to precepting nursing students.

### 2.3. Sample and Sampling Procedure

The study employed total population sampling by involving potentially every member of the accessible population eligible for inclusion in the study. All the clinical placement sites–Cape Coast Teaching Hospital, Cape Coast Metropolitan Hospital, Ankaful Psychiatric Hospital, University of Cape Coast Hospital, and Ewim Polyclinic were included in the study. At the time of the study, information gathered at the human resource and nursing administration of all the institutions showed that the following number of nurses and midwives met the eligibility criteria for inclusion in the study. These are Ankaful Psychiatric Hospital-165, University of Cape Coast Hospital-52, Metropolitan Hospital-35, Cape Coast Teaching Hospital-282, and Ewim Polyclinic-19. Although the study anticipated a total of 553 nurses and midwives for inclusion in the study, those who actually participated were 442, with a response rate of 79.9%. The reasons for nonparticipation included lack of general interest and busy schedules both at work and home as few participants had the option to complete the questionnaires off-site.

### 2.4. Data Collection Instrument

A questionnaire was developed based on literature on preceptorship in nursing [14, 15, 24–26]. The questionnaire comprised the following subscales; knowledge about preceptorship, perceived motivators for performing the preceptor role, perceived inhibitors to precepting nursing students, and the support preceptors need to effectively engage in the preceptorship role. In this study, knowledge was defined as the information or understanding that nurses and midwives have regarding preceptorship. Perceived motivators referred to things that nurses and midwives' perceived as factors that encouraged them to take up the preceptor role. Perceived inhibitors referred to nurses and midwives' perception of the factors that discouraged or impeded their participation in the preceptor role. Support referred to the availability of the desired resources for effective performance of the preceptor role.

The knowledge subscale comprised ten items on what preceptorship is or the definition of preceptorship adapted from literature [14, 27] and the participants were asked to indicate their knowledge of preceptorship by responding either “Yes,” “No,” or “Don't Know.” Also, the participants responded to the question, “what are the perceived motivators to precepting nursing students?” The perceived motivators subscale comprised 17 items adapted from literature [28] and was measured on a four-point Likert scale. The participants were asked to indicate their level of agreement or disagreement to the statements constituting the subscale by either responding strongly agree (SA), agree (A), disagree (D), or strongly disagree (SD).

The participants also responded to the question, “what perceived factors will hinder you from performing the preceptor role?” The perceived barriers subscale comprised 15 items, all measured on a four-point Likert scale. These items were adapted from previous studies [24, 25]. The participants were asked to indicate their level of agreement or disagreement to the statements constituting the subscale by either responding strongly agree (SA), agree (A), disagree (D), or strongly disagree (SD).

The support subscale had eight items adapted from literature [29]. The participants responded to the question, “what support do preceptors need to effectively perform their roles?” The participants were required to state their level of agreement or disagreement to the items on the subscale by responding either agree or disagree.

The study considered the following sociodemographic information of the participants; gender, age, professional rank, work experience, and duration of precepting nursing students.

Face validity was ensured by careful review by two experts in the field of nursing with in-depth experience in preceptorship. These experts were nurse leaders who had extensive experience in precepting nursing students for over two decades. Also, efforts were made to ensure that the questionnaire items reflected the objectives of the study. A pretest was conducted with 30 nurses in a nearby health facility to ensure that the questions were clear and understandable. The negatively worded items were reverted and the Cronbach's coefficient of reliability was used to determine the reliability of the Likert-scale items while Kuder and Richardson's statistics (KR-20) was used to assess the internal consistency of the items with dichotomous options. Therefore, KR-20 statistics was used to determine reliability of the items on the knowledge and support subscales. Cronbach's alpha was used for the items on the perceived motivators and perceived barrier subscales because these were on a Likert scale. The study yielded the following reliability coefficients for the different subscales; knowledge = 0.714, perceived motivators = 0.810, perceived barriers = 0.825, and support = 0.720. According to Bryman [30], reliability coefficient of 7.0 is acceptable for new measures.

### 2.5. Data Collection

Five graduate nurses were recruited and trained as research assistants to collect relevant data for the study. The training covered how the items on the questionnaire should be answered. In the various wards, eligible participants were approached and those willing to participate were included in the study. A thorough explanation about the study was provided and written informed consent was obtained from each participant. To ensure privacy, participants were allowed to answer the questionnaires at the nurses' lounge/room after they had finished their day's activities on the ward. The questionnaires did not capture any personal identifying information on the participants thereby ensuring anonymity. Consequently, the data obtained could not be linked to any of the participants. Also, 12 participants who were unable to fill the questionnaire in the ward were allowed to complete it off-site, and they returned it to the research assistant within the period of data collection. The data collection exercise took approximately six weeks from November to December, 2019. In all, 442 nurses and midwives participated in the study.

### 2.6. Data Analysis

The data were analyzed using the Statistical Package for Social Sciences version 21.0. The statistics used included frequency counts, percentages, exploratory factor analysis, and point biserial correlation. Prior to the analysis, scores for negatively worded items were reversed. Specifically, to assess the knowledge level of nurses and midwives on preceptorship, the aggregate score for knowledge test was determined and categorized into low, moderate, and high with the following scores. Scores of 59% and below constituted low knowledge, between 60% and 79% were categorized as moderate knowledge, and above 80% categorized as high knowledge. Exploratory factor analysis was used to elucidate how the different items on the perceived motivator and inhibitor subscales relate to one another and to determine the main perceived motivators and inhibitors to precepting nursing students within the Cape Coast Metropolis. The support preceptors needed to effectively engage in the preceptorship role subscale was analyzed using frequency counts and percentages. The relationship between perceived motivators, perceived inhibitors, and the intention to precept nursing students in the near future were determined using point biserial correlation.

## 3. Results

### 3.1. Sociodemographic Characteristics of the Participants

The results show that 62.7% (*n* = 277) of the participants were females while 37.3% (*n* = 165) were males. The age of the participants ranged from 27 to 56 years, with a mean of 32.48 and a standard deviation of 5.11. Furthermore, 12.0% (*n* = 53) of the participants were staff nurses/midwives, 47.3% (*n* = 209) were senior staff nurses/midwives, 27.1% (*n* = 120) were nursing officers/midwifery officers, 8.1% (*n* = 36) were senior nursing/midwifery officers/, 5.0% (*n* = 22) were principal nursing/midwifery officers, and 0.5% (*n* = (2) were deputy directors for nursing services. The involvement of senior nurses, midwives, and managers in preceptorship is expected in the Ghanaian context since they have vast experience in mentoring novice nurses and midwives.

### 3.2. Training, Intention, and Duration of Practicing Nursing and Preceptorship

The majority of the participants, 91.9% (*n* = 407), indicated that they had not been trained to precept nursing students while 8.1% (*n* = 36) have had training. Nonetheless, 88.2% (*n* = 390) had the intention to precept nursing students in the near future while 11.8% (*n* = 52) did not have any intention to precept students. Regarding how long the participants had practiced nursing/midwifery, the results indicate that participants had experience ranging from 3 to 30 years, with a mean of 6.71 and a standard deviation of 4.47. Also, some of the participants had been precepting students from 3 to 19 years with a mean of 4.61 and a standard deviation of 3.03.

### 3.3. Knowledge of Participants about Preceptorship

Regarding the level of knowledge of the participants on preceptorship, 91.2% (*n* = 404) had high knowledge of preceptorship, 8.4% (*n* = 37) had moderate knowledge, and only 0.4% (*n* = (2) had low knowledge. Specifically, Table 1 shows that 97.1% (*n* = 429) opined that preceptorship is about teaching students while on clinical placement. Significant proportions of the participants, 96.8% (*n* = 428), viewed preceptorship as helping students meet the objectives for the placement while 96.8% (*n* = 428) also viewed it as helping students to demonstrate current knowledge during placement. Furthermore, 94.6% (*n* = 418) viewed it as helping students to manage their clinical hours effectively. However, 25.8% (*n* = 114) of the participants conceptualized preceptorship to mean encouraging the student to obey the preceptor all the time while 48.9% (*n* = 216) indicated that preceptorship focuses on allowing students to perform preferred tasks without interference.

### 3.4. Perceived Motivators for Precepting Nursing Students

Table 2 presents the descriptive statistics on the various items on the perceived motivators subscale. A high proportion of the participants, 56.3% (*n* = 249) strongly agreed and 43.0% (*n* = 190) agreed to the statement that their perceived motivation for precepting the nursing students were to enhance student skills. Again, 56.3% (*n* = 249) strongly agreed and 41.9% (*n* = 185) agreed to the assertion of building students' confidence. However, 23.1% (*n* = 102) disagreed to the assertion of helping students to acquire resources for clinical learning as a perceived motivator for precepting them. Also, 18.3% (*n* = 81) and 13.8% (*n* = 61) disagreed to the assertion accounting for students from diverse backgrounds, and helping nursing students' network effectively as perceived motivators for engaging in the preceptorship role, respectively. Fourteen-point three percent (*n* = 63) disagreed to the assertion of precepting students to gain professional recognition while 15.8% (*n* = 70) disagreed to the assertion that they precept students because they had a similar experience.

Furthermore, the results in Table 3 present the perceived motivational factors to precepting nursing students with exploratory factor analysis. Three perceived motivator factors had eigenvalues greater than 1 so the final factor solution represented 49.27% of the variance in the data. The three important perceived motivator factors to precept nursing students were the learning and professional needs of students, helping students to develop skills, and experience and professional recognition of preceptorship with eigenvalues of 5.84, 1.44 and 1.09, correspondingly that accounted for 34.36%, 8.47% and 6.44%, of the variance in the data, respectively.

Specifically, items such as opportunity to help students set career goals, stimulate creativity at the workplace, establish life/work balance, account for students from diverse backgrounds, and acknowledge nursing students' contributions were dominant in explaining the learning and professional needs of students as a perceived motivator factor for precepting nursing students.

With regards to helping students develop skills as a perceived motivator factor, items such as the opportunity to meet the objectives of the students, establish a healthy relationship with the students, listen to students effectively, and provide constructive feedback were more pronounced. Moreover, regarding the experience and professional recognition of preceptors as perceived motivator factor, the opportunity to gain professional recognition was a more distinct factor.

### 3.5. Perceived Inhibitors for Successfully Performing the Preceptor Role

Table 4 presents the descriptive statistics on the various items on the perceived inhibitors to successfully performing the preceptor role subscale. From the table, 26.0% (*n* = 115) strongly agreed and 39.4% (*n* = 174) agreed to the assertion that they do not get support from faculty when students are on placement. Almost a quarter, 24.2% (*n* = 107), strongly agreed and 42.3% (*n* = 187) agreed to the assertion that they have a primary responsibility to provide patient care. Nonetheless, 48.9% (*n* = 216) strongly disagreed and 24.0% disagreed with the assertion that they are not well prepared to precept nursing students. Similarly, 47.5% (*n* = 210) strongly disagreed and 24.7% (*n* = 109) disagreed to the assertion that they are not confident enough to precept students.

The results in Table 5 present the perceived inhibitors to successfully performing the preceptor role from the exploratory factor analysis. Three perceived barriers had eigenvalues greater than 1, and the final factor solution represented 51.56% of the variance in the data. The main perceived barriers to engaging in the preceptorship role were lack of preparation for the role, lack of support from faculty and managers, and additional burden with eigenvalues of 5.21, 1.33, and 1.19 that accounted for 34.75%, 8.84%, and 7.95% of the variance in the data, respectively.

Furthermore, items including “I am not confident enough to precept nursing students,” “I am not well prepared to precept nursing students, and “I see precepting as a challenging task” were dominant in explaining the lack of preparation for the preceptorship role. Similarly, items such as “I do not get support from my manager,” “I do not get support from faculty when students are on placement,” and “I do not have enough teaching and learning resources to teach students were more dominant in explaining the lack of support as a perceived barrier. In the same way, items including “I have a primary responsibility to provide patient care,” and “I see precepting as an additional demand” were more distinct in explaining the additional burden as a perceived barrier to performing the preceptor role by the participants.

### 3.6. Support Preceptors Need to Successfully Perform Their Roles

Table 6 presents the results on the items on the support preceptors needed to successfully perform their roles. From the table, the majority of the participants, 98.4% (*n* = 435), agreed to the assertion that in-service training on preceptorship should be organized for preceptors. Also, 95.7% (*n* = 423) and 95.0% (*n* = 420) agreed to the assertions that training on clinical teaching and support from experienced preceptors on how to manage role, respectively. Again, 76.2% (*n* = 337) agreed that the preceptorship role should be recognized as a criterion for promotion while 23.8% (*n* = 105) disagreed with this assertion.

### 3.7. Relationship between Perceived Motivators, Perceived Inhibitors, and Intention of Precepting Nursing Students in the near Future

Regarding the perceived motivators, the results of the correlation show a significant weak positive relationship between helping students to develop skills and intention to precept nursing students in the near future (*r* = 0.161, *p*=0.001). There was also a significant weak positive correlation between learning and professional needs of students and intention (*r* = 0.102, *p*=0.032). The results further indicate a significant strong positive correlation between experience and professional recognition and intention to precept nursing students in the near future (*r* = 0.99, *p*=0.037). With regard to the perceived inhibitors, there was no statistically significant relationship between additional burden (*r* = −0.034, *p*=0.470), lack of support (*r* = −0.005, *p*=0.916), and lack of preparation (*r* = −0.059, *p*=0.216), and intention to precept nursing students in the near future.

## 4. Discussion

### 4.1. Knowledge of Participants about Preceptorship

Nurses and midwives engage in complex and multifaceted roles in undergraduate nursing education [30]. Effective performance of these roles requires adequate knowledge of preceptorship to assist students in acquiring the expected competencies. The findings of this study indicate that the nurses and midwives sampled had a high knowledge of preceptorship even though the majority had not been formally trained on preceptorship. A plausible explanation could be that knowledge test basically focused on the definition of preceptorship. It could also be due to the fact that the test items had few diverters, which might have influenced the participants to obtain high scores. In addition, they may have read about preceptorship, have had an experience with a preceptor, or even functioned as a preceptor. A study conducted in Kenya found the preceptors to be knowledgeable on preceptorship though most of them acquired this knowledge from experience and role modeling [14]. It is worth noting that over 90% of the participants viewed preceptorship as teaching students while on placement, helping students meet their objectives, creating conducive environment to facilitate learning, and helping students manage their clinical hours effectively. A previous study affirmed that the preceptor facilitates the development of practical skills, professional socialization, report and documentation, prioritization, communication, and planning of daily activities [28]. Surprisingly, 48.9% of the participants stated that preceptorship is “allowing students to perform the preferred task without interference” while 25.8% viewed preceptorship as “encouraging the student to obey the preceptor at all times.” These findings demonstrate critical gaps in knowledge as these approaches to preceptorship may not encourage critical thinking among students. Preceptorship demands that students practice under direct supervision at all times. The preceptor also shares experience and knowledge with students to facilitate the acquisition of clinical competencies and critical thinking skills [12].

### 4.2. Perceived Motivators for Precepting Nursing Students

The findings further suggest that the important perceived motivational factors for precepting nursing students were the learning and professional needs of students, helping students to develop skills, and experience and professional recognition of preceptorship. The desire of the nurses and midwives in this study to precept nursing students could be that they had similar experiences. Findings also suggest that preceptors are concerned with equipping the next generation of nurses and midwives with the requisite competencies to enable them to function effectively. They are also interested in gaining professional recognition from performing that role which is consistent with a study conducted in Ghana [13]. An earlier work reported the need to give back to the profession as the main motivating factor for performing the preceptor role [17]. Nonetheless, a study conducted among nurse practitioner students in a high-income country found relationships with faculty, adjunct faculty status, and access to free continuing professional development programmes as the most important motivators for preceptors [31]. Other incentives that could persuade professional nurses and midwives to precept students include gaining credit for recertification, professional responsibility, opportunities to learn, and forming relationship with faculty or students [13, 32]. It is evident that by engaging in their assigned role, preceptors tend to gain personal rewards of being role models, develop knowledge and reenergize self in nursing practice, and even develop interest in a stimulating career in nursing education in other settings [32]. A well-motivated preceptor will, therefore, build students' confidence and facilitate the achievement of clinical competencies in line with the learning needs of the students [33].

### 4.3. Perceived Inhibitors to Successfully Performing the Preceptor Role

Furthermore, the study identified lack of preparation for role, lack of support from faculty and managers, and additional burden as the main perceived barriers to engaging in the preceptorship role. The findings imply that these impediments need to be overcome by nurses and midwives to effectively perform the preceptor role. Since preceptorship is pivotal in the educational development of nurses, adequate preparation is essential for the smooth transition into the preceptor role. Many nurses and midwives are unwilling to undertake the role due to perceived lack of skill to manage students [19]. From this study, additional factors that explained the lack of preparation from the perspectives of the nurses and midwives sampled were lack of confidence, readiness, planning, and perception of preceptorship being a challenging task. Distress accompanying the teaching role was cited as a major barrier in an earlier study [19]. This demands some flexibility in the selection and training of preceptors to ensure that nurses and midwives with the desire and clinical competence for the role are trained and supported to enhance students' learning outcomes and bridge the theory-practice gap. Again, the training will enable preceptors to acquire more information and skills about the concept of preceptorship, approaches to clinical teaching and learning, reflective practice and clinical reasoning [15].

Similarly, the participants sampled reported a lack of support from faculty and managers when students are on clinical placements. In addition, lack of teaching and learning resources and training were cited as perceived inhibitors to successfully performing the role. These findings require faculty to closely collaborate and establish a healthy relationship with preceptors by communicating the learning outcomes of the students to preceptors. Also, it is imperative that clinical nurse managers or leaders support preceptors to perform their fundamental responsibility of caring for patients in addition to precepting students. A phenomenological study conducted in Iran also reported a lack of support for preceptors [16]. This suggests that the problem of preceptors demanding support is cross-national in nature, which requires attention of nurse educators and managers. However, in a high-income setting like Texas, nursing faculty provides extensive support to preceptors to guide students' learning by orienting students and preceptors to the course guide and policies as stated in the curriculum. They also clearly state the role the preceptor is supposed to play for the specific course, establish means of communication to discuss students' progress, and assign a grade for the course [32].

Furthermore, the nurses and midwives who participated in this study viewed preceptorship as an additional burden. A possible explanation is that some of the participants felt they had a primary responsibility to provide patient care and as such they either have little or no time to precept students while others felt overwhelmed with the preceptor role. Currently, the method of preceptorship whereby the preceptors are fully engaged by a healthcare agency and thus have a fundamental role to play in the agency does not allow them to have sufficient time for students during clinical placement. Also, students' numbers keep on increasing across nursing programmes, yet there are only limited clinical sites for placements. Consequently, preceptors end up experiencing burnout and students too may not meet their clinical objectives [15]. This calls for other approaches to precepting nursing students to ensure the acquisition of clinical competencies and delivery of quality nursing care.

### 4.4. Support Preceptors Need to Successfully Perform Their Roles

The findings further indicate that over 90% of the nurses and midwives sampled reported that in-service training, training on clinical teaching, and support from experienced preceptors on how to manage will facilitate effective performance of the preceptor role. These findings affirm the need for potential preceptors to have adequate training on preceptorship to sharpen their knowledge and skills. This is essential as not all professional nurses and midwives are good clinical teachers. Even those with a strong desire to coach nursing students need to be trained on the whole process of preceptorship, clinical teaching and reasoning, as well as reflective practice to enable them adopt evidence-based strategies that could maximize the learning experiences of nursing students [14, 32]. Likewise, healthcare organizations, educational institutions, and managers need to support preceptors and preceptees by providing adequate resources for clinical training and show keen interest in the training of preceptors. It is imperative that experienced preceptors also assist the novice ones to successfully transit into the preceptor role.

Also, some of the nurses and midwives in this study agreed that preceptorship should be recognized as a criterion for promotion. It is believed that when the professional bodies and healthcare organizations and agencies view it as one of the criterion for promotion to a higher rank in the nursing profession, more nurses and midwives will be extra committed to functioning in the role, thereby shaping and transmitting appropriate culture and values of the nursing profession into the next generation. In achieving this, a component of the annual appraisal for nurses and midwives could focus on preceptorship to enable those with the desire to gradually work at accomplishing that competency.

### 4.5. Relationship between Perceived Motivators, Perceived Inhibitors, and Intention of Precepting Nursing Students in the near Future

The findings further show a strong positive relationship between experience and professional recognition and intention to precept nursing students in the near future (*r* = 0.99, *p*=0.037). This suggests that when the preceptorship engagements of nurses and midwives with experience are recognized professionally, they will have the intention to precept nursing students in the near future. This recognition will serve as a form of incentive for their role. Hence, there should be formal ways of evaluating and certifying preceptorship activities to enable them gain recognition for their actions. It is interesting to note that in Malawi and Texas, there are well-established guidelines for preceptorship that allow preceptors to gain some rewards toward their professional development [32, 33]. This study observed a weak positive relationship between helping students to develop skills, and learning and professional needs of students, with intention to precept students in the near future. More empirical work is necessary to clarify this relationship. In relation to the perceived inhibitors, the current study did not find any relationship between the additional burden, lack of support, and lack of preparation and intention to precept nursing students in the near future. This means that as long as these impediments hinged around preceptorship, participants may not have the intention of precepting nursing students in the near future. It is, therefore, crucial that health training institutions and all important stakeholders in nursing education become intentional about these perceived inhibitors so as to curtail them. The findings of this study can be generalized to the study setting and beyond.

## 5. Conclusions

Preceptorship is integral in nursing education programmes. This study has highlighted the fact that most nurses and midwives are knowledgeable about preceptorship and have the intention of precepting nursing students in the near future. The underlying perceived motivation that will enable these professionals to engage in the preceptorship role include the learning and professional needs of the students, the ability to help students to develop skills, and experience and formal recognition of preceptorship. It is important to note that experience and formal recognition of preceptorship may enable more nurses and midwives to have the intention of precepting nursing students in the near future. However, certain factors could inhibit these professionals from effectively performing the preceptorship role which need to be addressed by identifying context-specific solutions to maximize the experiences of nursing students.

## Figures and Tables

**Table 1:** Descriptives on the knowledge of nurses and midwives on preceptorship (*N* = 442).

Knowledge statement	Yes frequency (%)	No frequency (%)	Don't know frequency (%)
Helping students meet their objectives for the placement	428 (96.8)	8 (1.8)	6 (1.4)
Teaching students while on clinical placement	429 (97.1)	9 (2.0)	4 (0.9)
Helping students demonstrate current knowledge during placement	428 (96.8)	3 (0.7)	11 (2.5)
Focusing on the learning needs of the students	407 (92.1)	28 (6.3)	7 (1.6)
Creating a conducive environment to facilitate learning at the placement site	404 (91.4)	23 (5.2)	15 (3.4)
Helping students manage their clinical hours effectively	418 (94.6)	17 (3.8)	7 (1.6)
Coaching and training competence in a life-long perspective	388 (87.8)	30 (6.8)	24 (5.4)
Assigning tasks to students while on clinical placement	395 (89.4)	43 (9.7)	4 (0.9)
Allowing students to perform preferred tasks without interference	216 (48.9)	211 (47.7)	15 (3.4)
Encouraging students to obey the preceptor at all times	114 (25.8)	304 (68.8)	24 (5.4)

**Table 2:** Descriptives on the perceived motivators to precepting nursing students (*N* = 442).

Perceived motivators	SA *f* (%)	A *f* (%)	D *f* (%)	SD *f* (%)
The opportunity to help students set career goals	156 (35.3)	239 (54.1)	44 (10.0)	3 (0.7)
The opportunity to stimulate creativity at the workplace	165 (37.3)	257 (58.1)	19 (4.3)	1 (0.2)
The opportunity to build confidence in nursing students	249 (56.3)	185 (41.9)	7 (1.6)	1(0.2)
The opportunity to acknowledge nursing students' contributions	149 (33.7)	267 (60.4)	24 (5.4)	2 (0.5)
The opportunity to account for students from diverse backgrounds	113 (25.6)	242 (54.8)	81 (18.3)	6 (1.4)
The opportunity to help nursing students acquire resources for clinical learning	84 (19.0)	243 (55.0)	102(23.1)	13 (2.9)
The opportunity to help nursing students network effectively	123 (27.8)	255 (57.7)	61 (13.8)	3 (0.7)
The opportunity to help nursing students establish a life/work balance	116 (26.2)	274 (62.0)	50 (11.3)	2 (0.5)
The opportunity to enhance the skills of nursing students	249 (56.3)	190 (43.0)	3 (0.7)	—
The opportunity to engage with students while on placement	180 (40.7)	232 (52.5)	27 (6.1)	3 (0.7)
The opportunity to develop strategies to meet the objectives of the students	218 (49.3)	212 (48.0)	12 (2.7)	—
The opportunity to establish a healthy relationship with students	174 (39.4)	254 (57.5)	13 (2.9)	1 (0.2)
The opportunity to listen to students effectively	187 (42.3)	233 (52.7)	19 (4.3)	3 (0.7)
The opportunity to provide constructive feedback	182 (41.2)	229 (51.8)	29 (6.6)	2 (0.5)
The opportunity to develop a trusting relationship with students	148 (33.5)	251 (56.8)	38 (8.6)	5 (1.1)
The opportunity to gain professional recognition through preceptorship	148 (33.5)	223 (50.5)	63 (14.3)	8 (1.8)
The opportunity to precept students as I had a similar experience	121 (27.4)	242 (54.8)	70 (15.8)	9 (2.0)

**Table 3:** Exploratory factor analysis on the perceived motivators to precepting nursing students.

Scale items	Loadings	Perceived motivator factors
*Component 1*		
The opportunity to help students set career goals	0.575	Learning and professional needs of students
The opportunity to stimulate creativity at the workplace	0.557	
The opportunity to acknowledge nursing students' contributions	0.523	
The opportunity to account for students from diverse backgrounds	0.582	
The opportunity to help nursing students acquire resources for clinical learning	0.582	
The opportunity to help nursing students network effectively	0.690	
The opportunity to help nursing students establish a life/work balance	0.622	

*Component 2*		
The opportunity to build confidence in nursing	0.576	Helping students to develop skills
The opportunity to enhance the skills of nursing students	0.660	
The opportunity to engage with students while on placement	0.647	
The opportunity to develop strategies to meet the objectives of the students	0.720	
The opportunity to establish a healthy relationship with students	0.711	
The opportunity to listen to students effectively	0.672	
The opportunity to provide constructive feedback	0.652	
The opportunity to develop a trusting relationship with students	0.502	

*Component 3*		
The opportunity to gain professional recognition	0.762	Experience and professional recognition of preceptorship
The opportunity to precept students as I had a similar experience	0.724	

**Table 4:** Descriptives on the perceived barriers to successfully perform the preceptor role (*N* = 442).

Perceived barriers	SA *f* (%)	A *f* (%)	D *f* (%)	SD *f* (%)
I do not have time to precept students	48 (10.9)	106 (24.0)	200 (45.2)	88 (19.9)
I see precepting as an additional demand	68 (15.4)	154 (34.8)	174 (39.4)	46 (10.4)
I have a primary responsibility to provide patient care	107 (24.2)	187(42.3)	121 (27.4)	27 (6.1)
I often have little time to work with students at the clinical setting	53 (12.0)	165 (37.3)	172 (38.9)	52 (11.8)
I feel overwhelmed with my role as a preceptor	47 (10.6)	112 (25.3)	223 (50.5)	60 (13.6)
I am not well prepared to precept students	31 (7.0)	89 (20.1)	216 (48.9)	106(24.0)
I do not get support from faculty when students are on clinical placement	115 (26.0)	174(39.4)	123 (27.8)	30 (6.8)
I do not have enough teaching and learning resources to teach students	90 (20.4)	171 (38.7)	129(29.2)	52 (11.8)
I do not get the necessary support from my manager	60 (13.6)	133 (30.1)	197 (44.6)	52 (11.8)
I have to precept too many students at the same time	77 (17.4)	165 (37.3)	166(37.6)	34 (7.7)
I am not able to plan for the precepting process	44 (10.0)	154 (34.8)	204 (46.2)	40 (9.0)
I have not been selected to precept students although I have the desire for it	92 (20.8)	143 (32.4)	167 (37.8)	40 (9.0)
I am not confident enough to precept students	16 (3.6)	58 (13.1)	210 (47.5)	158(35.7)
I see precepting as a challenging task	32 (7.2)	115 (26.0)	186 (42.1)	109(24.7)
I have not been trained to precept nursing students	86 (19.5)	173 (39.1)	124 (28.1)	59 (13.3)

**Table 5:** Exploratory factor analysis on perceived barriers to successfully perform the preceptor role.

Scale items	Loadings	Perceived barriers
*Component 1*		
I am not confident enough to precept students	0.809	Lack of preparation for role
I am not well prepared to precept students	0.706	
I see precepting as a challenging task	0.695	
I am not able to plan for the precepting process	0.569	

*Component 2*		
I do not get the necessary support from my manager	0.678	Lack of support
I do not get support from faculty when students are on clinical placement	0.659	
I do not have enough teaching and learning resources to teach students	0.629	
I have not been selected to precept students although I have the desire for it	0.609	
I have to precept too many students at the same time	0.581	
I have not been trained to precept nursing students	0.470	

*Component 3*		
I do not have time to precept students	0.534	Additional burden
I see precepting as an additional demand	0.635	
I have a primary responsibility to provide patient care	0.729	
I often have little time to work with students at the clinical setting	0.590	
I feel overwhelmed with my role as a preceptor	0.599	

**Table 6:** Support preceptors need to successfully perform their roles *N* = 442.

Type of support	Agree *f* (%)	Disagree *f* (%)
In-service training on preceptorship	435 (98.4)	7 (1.6)
Training on clinical teaching	423 (95.7)	19 (4.3)
Training on reflective practice and clinical reasoning	414 (93.7)	28 (6.3)
Support from experienced preceptor on how to manage the role	420 (95.0)	22 (5.0)
Remuneration for the preceptor role	401 (90.7)	41 (9.3)
Higher education opportunities to equip preceptors	392 (88.7)	50 (11.3)
Recognition of role as a criterion for promotion	337 (76.2)	105 (23.8)
Recognition of role as evidence for renewal of professional license	372 (84.2)	70 (15.8)

## Data Availability

The datasets used and/or analyzed during the current study are available from the corresponding author on reasonable request.
